# Benefits to Speech Perception in Noise From the Binaural Integration of Electric and Acoustic Signals in Simulated Unilateral Deafness

**DOI:** 10.1097/AUD.0000000000000252

**Published:** 2015-05-04

**Authors:** Ning Ma, Saffron Morris, Pádraig Thomas Kitterick

**Affiliations:** 1 Medical Research Council Institute of Hearing Research, University Park, Nottingham, United Kingdom; 2 National Institute for Health Research Nottingham Hearing Biomedical Research Unit, Ropewalk House, Nottingham, United Kingdom; and 3 Otology and Hearing Group, Division of Clinical Neuroscience, School of Medicine, University of Nottingham, Nottingham, United Kingdom.

**Keywords:** Binaural hearing, Binaural integration, Cochlear implantation, Single-sided deafness, Speech perception, Unilateral deafness

## Abstract

**Objectives:**

This study used vocoder simulations with normal-hearing (NH) listeners to (1) measure their ability to integrate speech information from an NH ear and a simulated cochlear implant (CI), and (2) investigate whether binaural integration is disrupted by a mismatch in the delivery of spectral information between the ears arising from a misalignment in the mapping of frequency to place.

**Design:**

Eight NH volunteers participated in the study and listened to sentences embedded in background noise via headphones. Stimuli presented to the left ear were unprocessed. Stimuli presented to the right ear (referred to as the CI-simulation ear) were processed using an eight-channel noise vocoder with one of the three processing strategies. An *Ideal* strategy simulated a frequency-to-place map across all channels that matched the delivery of spectral information between the ears. A *Realistic* strategy created a misalignment in the mapping of frequency to place in the CI-simulation ear where the size of the mismatch between the ears varied across channels. Finally, a *Shifted* strategy imposed a similar degree of misalignment in all channels, resulting in consistent mismatch between the ears across frequency. The ability to report key words in sentences was assessed under monaural and binaural listening conditions and at signal to noise ratios (SNRs) established by estimating speech-reception thresholds in each ear alone. The SNRs ensured that the monaural performance of the left ear never exceeded that of the CI-simulation ear. The advantages of binaural integration were calculated by comparing binaural performance with monaural performance using the CI-simulation ear alone. Thus, these advantages reflected the additional use of the experimentally constrained left ear and were not attributable to better-ear listening.

**Results:**

Binaural performance was as accurate as, or more accurate than, monaural performance with the CI-simulation ear alone. When both ears supported a similar level of monaural performance (50%), binaural integration advantages were found regardless of whether a mismatch was simulated or not. When the CI-simulation ear supported a superior level of monaural performance (71%), evidence of binaural integration was absent when a mismatch was simulated using both the Realistic and the Ideal processing strategies. This absence of integration could not be accounted for by ceiling effects or by changes in SNR.

**Conclusions:**

If generalizable to unilaterally deaf CI users, the results of the current simulation study would suggest that benefits to speech perception in noise can be obtained by integrating information from an implanted ear and an NH ear. A mismatch in the delivery of spectral information between the ears due to a misalignment in the mapping of frequency to place may disrupt binaural integration in situations where both ears cannot support a similar level of monaural speech understanding. Previous studies that have measured the speech perception of unilaterally deaf individuals after CI but with nonindividualized frequency-to-electrode allocations may therefore have underestimated the potential benefits of providing binaural hearing. However, it remains unclear whether the size and nature of the potential incremental benefits from individualized allocations are sufficient to justify the time and resources required to derive them based on cochlear imaging or pitch-matching tasks.

## Abstract 2

The current study simulated cochlear implant use in normal-hearing listeners to examine whether speech information from a normal-hearing ear can be integrated with the spectrally and temporally degraded information that an implant provides. The study also assessed whether binaural integration is disrupted by a mismatch in the delivery of spectral information between the ears. Evidence of binaural integration and associated benefits to speech perception were observed even when a mismatch was introduced. It is unclear whether additional binaural benefits may be obtained by minimizing mismatch between the ears through the use of individualized frequency-to-electrode allocations.

## INTRODUCTION

Individuals with a single-sided deafness (SSD), who have severe to profound hearing loss in one ear and normal or near-normal hearing in the other ear, experience difficulty understanding speech in background noise (McLeod et al. 2008). When speech and background noise are presented at the same level, individuals with SSD hear only about 30% to 35% of the conversation (Christensen et al. 2010). Such difficulties may lead to significant communication handicaps that compromise the quality of life of these unilaterally hearing-impaired individuals (Noble & Gatehouse 2004; Wie et al. 2010). Severe to profound unilateral hearing loss in children may present them with particular difficulties in general group activities, leading to delays in development of speech and language, and affecting their academic performance and educational progress (Bess & Tharpe 1986; Tharpe & Sladen 2008).

To date, individuals with permanent SSD have limited treatment options. A contralateral routing of signals hearing aid or a bone conduction hearing aid can be used to route signals arriving at the deaf ear to the normal-hearing (NH) ear via air or bone conduction, respectively. These solutions improve access to sound by overcoming the acoustic shadow cast by the head that would otherwise attenuate sounds located on the deafened side (Pumford 2005). A limitation of these systems is that they rely solely on the hearing ear and do not restore input to the deafened ear. As a consequence, these systems do not alleviate the many communication handicaps that individuals with SSD experience, which relate to the fact that they are functioning with unilateral auditory input (Bishop & Eby 2010).

The provision of binaural hearing through cochlear implantation (CI) can improve speech perception in challenging listening conditions relative to monaural hearing alone (Köbler & Rosenhall 2002; Schleich et al. 2004; Litovsky et al. 2009). When speech and noise are spatially separated, a binaural benefit can be achieved simply by listening to whichever ear has the more favorable signal to noise ratio (SNR) regardless of which side of the head the speech is located (“better ear” effect). In NH listeners, as well as in a subset of CI users, binaural benefit can also be gained by integrating the information received at the two ears. When speech and noise are spatially separated, access to a second ear with a less-favorable SNR can help distinguish speech from noise by providing additional (albeit degraded) information about the signal and also the noise (“squelch” effect). Binaural benefit may also be gained by exploiting redundancy in two similar copies of the original signals such as when speech and noise are spatially coincident (“summation” effect).

CI has been investigated as a potentially effective method for providing binaural hearing in individuals with SSD (Vermeire & Van de Heyning 2009; Arndt et al. 2011; Hassepass et al. 2013) and those with highly asymmetric hearing losses (Firszt et al. 2012a). The primary benefits to speech perception from using a CI reported by these studies relate to better-ear effects rather than the binaural integration effects of summation and squelch. Vermeire and Van de Heyning (2009) compared speech-reception thresholds (SRTs) in nine patients with SSD 1 year after implantation with their implant turned on and off. SRTs were significantly lower (better) with the implant turned on when speech was presented on the side of the implant and noise was presented from the front, compatible with a better-ear effect. However, when noise was presented on the implanted side and speech in front, turning on the implant had no significant effect. A similar pattern of results was reported by Arndt et al. (2011) who measured SRTs in 11 SSD patients before and 6 months after implantation. SRTs improved significantly after implantation when speech was presented 45° toward the CI and noise at 45° toward the NH ear. However, SRTs did not change after implantation when noise was presented toward the CI and speech toward normal ear. Taken together, the existing evidence suggests that individuals with SSD may derive benefit from a CI when listening to speech in noise by attending to whichever ear has the more favorable SNR rather than by integrating information from the two ears.

The lack of evidence for binaural integration may be due in part to how SRTs have been measured. Previous studies have presented speech and noise from loudspeakers positioned on different sides of the head to create differences in SNR between the ears using the head’s acoustic shadow (Vermeire & Van de Heyning 2009; Arndt et al. 2011; Hassepass et al. 2013). However, there are substantial differences in the capacities of an implanted ear and a nonimplanted ear to support speech understanding in noise. On the same task, an NH ear can support accurate speech understanding even at negative SNRs, whereas speech understanding with an implanted ear alone can degrade even at SNRs well above 0 dB (Donaldson et al. 2009). Thus, a relatively large difference in SNR (>6 dB) can be necessary to achieve equivalent monaural performance levels in the implanted and nonimplanted ears of the same individual (Firszt et al. 2012b). As a result, many of the spatial configurations of speech and noise adopted in previous studies may have failed to overcome the large disparity in monaural performance between the ears such that listening to the NH ear alone was an effective and reliable strategy to maximize speech understanding.

It is also possible that the integration of information from the implanted and the NH ears of individuals with SSD is impaired by a mismatch in the delivery of spectral information between the ears. In an implanted ear, spectral information is unlikely to be delivered to the cochlear site with matching characteristic frequency as the frequency-to-place mapping is rarely based on the known position of the electrode array (Vaerenberg et al. 2014). Yoon et al. (2013) examined the effects of inducing a spectral mismatch between two implanted ears on speech perception in noise. NH individuals were presented with simulations of listening with two CIs, one in each ear. The implants either had identical frequency-to-place mappings (matched) or different mappings (mismatched). The perceived locations of speech and noise stimuli were varied to measure the binaural effects of summation and squelch. With the matched simulations, a significant beneficial effect of squelch was found when listening binaurally compared with listening monaurally. However, performance was impaired significantly when listening binaurally to the mismatched simulations compared with listening monaurally. It is unclear whether the lack of evidence for the binaural integration in individuals with SSD may be due, at least in part, to the presence of a spectral mismatch between their implanted ear and their NH ear.

The aims of the present study were to (a) measure the capacity of listeners to integrate speech information from an NH ear and a vocoder simulation of an implanted ear; and (b) investigate the impact of a mismatch in the delivery of spectral information between the two ears on binaural integration when listening to speech in noise. Simulations of listening with a CI in one ear and a contralateral NH ear were constructed to vary the degree to which the delivery of spectral information differed between the ears. The SNRs at the two ears were controlled independently to avoid an overdependence on the NH ear. Based on findings from CI users with limited residual hearing, it was expected that some evidence for the ability to integrate information between the two ears would be observed but that introducing a mismatch between the ears would disrupt integration and impair speech understanding.

## MATERIALS AND METHODS

### Power Calculation

A pilot study was conducted to estimate the variability in performance that would be observed on the sentence test used throughout this study. The results suggested a within-subject standard deviation of around 8 percentage points. The present study was powered to detect within-subject effects of at least this size, that is, effects of 1 standard deviation or larger. To achieve a one-tailed power of 0.8 at α = 0.05 required at least eight participants (Faul et al. 2007).

### Participants

Eight NH paid volunteers (age range 20 to 26 years, 3 males) participated in the main experiment and 12 (age range 18 to 29 years, 4 males) participated in an additional experiment. All were native speakers of British English and reported no impairments in their hearing or general health. Participants gave written informed consent, and the study was approved by the ethics committee of the School of Psychology, University of Nottingham.

### Stimuli

Sentences were selected from a British English recording of the Coordinate Response Measure (CRM) corpus (Kitterick et al. 2010). CRM sentences consist of a call-sign and a color-number co-ordinate embedded within a carrier phrase (Moore 1981). An example sentence is “Ready BARON go to GREEN FIVE now.” The sentences were constructed from the factorial combination of eight call-signs (“Arrow,” “Baron,” “Charlie,” “Eagle,” “Hopper,” “Laker,” “Ringo,” “Tiger”), four colors (red, white, blue, green), and the numbers from 1 to 8 to create a corpus of 256 sentences. The sentences were spoken by a single male talker with an average duration of 2.6 sec and were recorded at a sample rate of 44.1 kHz with 16 bits of quantization.

A speech-shaped noise was derived from the long-term average spectrum of the 256 sentences spoken by the same male talker. The average spectrum was estimated from the sentence materials using 4096-sample (93-msec) Hann windows with an overlap of 50%. The noise was generated by summing sine waves with random phase at 0.5-Hz intervals whose amplitude was determined from the estimated spectrum by linear interpretation.

### Signal Processing

The signals presented to each ear were either unprocessed or processed to approximate the spectral and temporal information conveyed by a CI.* The processing scheme comprises six steps: (1) The input signal was split into 8 adjacent spectral channels using zero-phase sixth-order elliptic band-pass filters (“analysis” filters); (2) The temporal envelope in each channel was extracted by half-wave rectification and low-pass filtering at 160 Hz using a zero-phase second-order elliptic filter; (3) The temporal envelope in each channel was used to modulate an independent sample of white noise of identical length to the input signal; (4) The resulting modulated noise in each channel was band-pass filtered using a zero-phase sixth-order elliptic filter (“output” filter); (5) The root mean square of the modulated and filtered noise in each channel was adjusted to match the root mean square of the input signal for that channel obtained from the band-pass filtering in step 1; (6) The eight modulated noises were summed to create the processed stimulus.

Table 1 lists the lower and upper edges of the analysis and output filters used to create the processed stimuli. The edge frequencies represent the 6-dB down points of each filter. The analysis filters were fixed regardless of the processing strategy and were selected to mimic the default analysis filters of the CI systems produced by Cochlear Ltd (Sydney, New South Wales, Australia). The output filters were varied to create three distinct processing strategies: *Ideal*, *Realistic*, and *Shifted*.

**TABLE 1.**
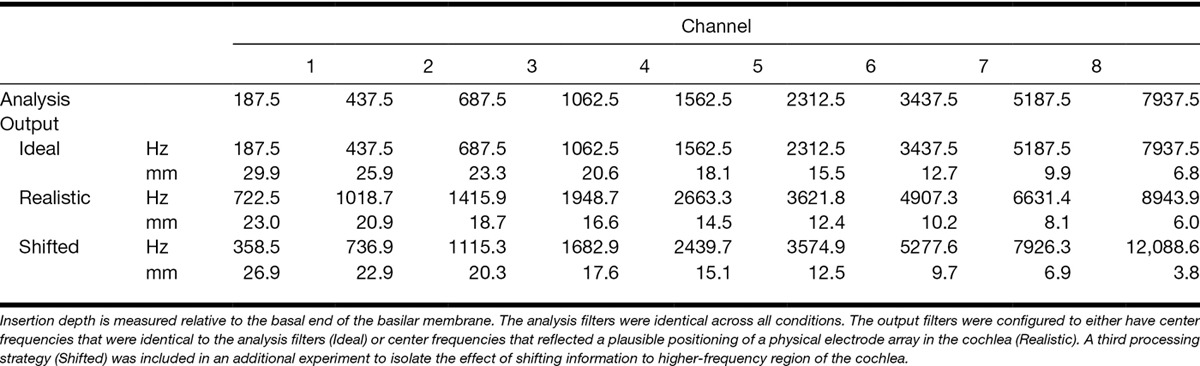
Lower and upper edge frequencies in Hz and in millimeters of insertion depth for the eight analyses and output filters used to construct the processing strategies that were applied to stimuli presented to the cochlear implant-simulation ear

For the Ideal strategy, the output filters were identical to the analysis filters. This strategy aligned the center frequency of each channel and the characteristic frequency of the place in the cochlea to which the channel information was delivered. It should be noted that the Ideal strategy as described here does not represent a strategy that is achievable in practice in CI users as it would require both a longer active electrode array length than is currently available and a deeper insertion than is typically desirable to avoid trauma to the cochlea. In the context of this study, Ideal refers to the theoretical ability to deliver spectral information over a wide range of frequencies to sites in the cochlea with similar characteristic frequencies. As such, the strategy ensured that the delivery of spectral information was matched between the NH and CI-simulation ears.

For the Realistic processing strategy, the output filters were adjusted to simulate a degree of misalignment in the mapping of frequency to cochlear place that could be expected to arise through the implantation of a commercially available electrode array. The length of the simulated electrode array† was based on the 17-mm active length of the Nucleus CI24RE(ST) implant (Cochlear Ltd). The positions of the eight adjacent output filters were also chosen to simulate an insertion depth of 23 mm from the basal end, approximating the median depth reported by surgeons for Nucleus implant systems (Yukawa et al. 2004). It also corresponds to a basal shift of 3 mm from a position midway along a typical 35-mm basilar membrane, which has been found to be sufficient to hinder binaural integration (Yoon et al. 2013). Thus, the Realistic strategy created a mismatch in the delivery of spectral information between the ears where the extent of the mismatch varied across frequency.

**Fig. 1.**
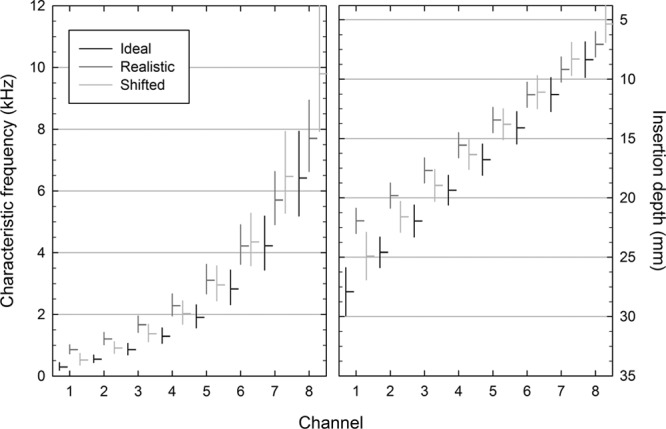
Graphical representation of the center frequencies (horizontal lines) and extent (vertical lines) of the output filters for the three processing strategies in terms of characteristic frequency (left panel) and insertion depth measured relative to the basal end of the basilar membrane (right panel).

The Realistic processing strategy has two notable features when compared with the Ideal strategy. First, the active length of the simulated array corresponds to a shorter (17 versus 23.1 mm) and more basal portion of the basilar membrane, effectively compressing and reducing the resolution of the available spectral information.‡ Second, the center frequencies of the analysis filters do not match those of the output filters, resulting in a misalignment between the frequency of the incoming information and the characteristic frequency of the cochlear place to which it is delivered. Any differences in performance observed between conditions using the Realistic and Ideal processing strategies could be attributed to either one or both these differences. A third processing strategy was therefore included (Shifted) that introduced a consistent misalignment in the mapping of frequency to place on the basilar membrane (3 mm) across all channels but which preserved the active length of the simulated electrode array compared with the Ideal condition. As a result, the Shifted strategy created a mismatch in the delivery of spectral information between the ears where the extent of the mismatch was similar across frequencies. The center frequencies and boundaries of the output filters for the three processing strategies are displayed in Figure 1.

### Procedure

Stimuli were generated digitally using MATLAB (MathWorks, Natick, MA, USA) and transmitted via a digital sound card (M-Audio, Cumberland, RI, USA) to a custom 24-bit digital-to-analog converter and headphone amplifier. Stimuli were presented over HD 600 headphones (Sennheiser, Wedemark, Germany). The digital levels of the sentences and the speech-shaped noise were calibrated to achieve a presentation level at the ear of 65-dB A-weighted SPL when either was presented in isolation. Calibration was performed using an artificial ear (B & K Type 4153) fitted with a flat-plate adaptor and a 0.5-in pressure field microphone (B & K Type 4192) connected to a sound level meter (B & K Type 2260).

On each trial, a CRM sentence was selected randomly from the corpus of 256 sentences. A segment of speech-shaped noise was generated so that its onset preceded that of the sentence by 1.25 sec and continued for 0.25 sec after the sentence had finished. The onset and offset of the noise were shaped in using 0.25-sec raised cosine amplitude transitions. The levels of the sentence and the noise were then adjusted to achieve the desired SNR; the noise was attenuated to achieve positive SNRs, and the speech was attenuated to achieve negative SNRs. Using this approach, the overall level of the combined stimulus was constrained to vary between 65 and 67 dB(A) SPL. Any further processing of the stimulus was dictated by the ear to which it was to be presented. Stimuli presented to the left ear of participants received no further processing. We will refer to the left ear as the NH ear. Stimuli presented to the right ear of participants were processed to simulate the information provided by a CI using one of the three processing strategies. We will refer to the right ear as the CI-simulation ear.

Stimuli were presented while participants were seated in a double-walled sound-isolated booth. Their task was to report the call-sign, color, and number key words in each sentence. The eight call-signs, four colors, and eight numbers were presented on a computer-controlled visual display. Participants indicated their response by selecting a single key word from each category using a computer mouse. A response was considered correct only when all three categories of key words were reported accurately.

To assess the extent to which listeners could integrate information from the two ears, it was first necessary to establish SNRs that produced known monaural performance levels for the NH and CI-simulation ears alone. These SNRs were established by estimating the monaural SRTs in each ear using an adaptive procedure (Levitt 1971). The SNR on the first trial of each procedure was chosen to produce an incorrect response based on pilot testing (−14 dB for the NH ear; −10 dB for the CI-simulation ear). The same sentence was then presented repeatedly while the SNR was increased in 2-dB steps until all three key words were identified correctly. A further 24 sentences were presented with the SNR on each trial determined by the accuracy of the previous response: the SNR was decreased by 2 dB after a correct response and increased by 2 dB after an incorrect response. The SRT was estimated by calculating the average of all SNRs at which the direction of change in SNR was reversed. The SRT was measured twice for each ear, and the average was used to determine the SNR at which a participant could accurately report all three key words in 50% of sentences using the NH ear or the CI-simulation ear alone. We will refer to these SNRs as NH50 and CI50, respectively.

The SNR at which a participant could accurately report all three key words in 71% of sentences using the CI-simulation ear alone was also estimated. The adaptive procedure was similar to that described previously, except that correct responses were required on two sequential trials to reduce the SNR by 2 dB. We will refer to the SNR corresponding to 71% correct as CI71. These monaural SNRs were subsequently used to control the level of accuracy attainable on a fixed-SNR version of the sentence test when using either ear alone.

The listening tests were administered across two sessions that were completed on different days. In the first session, stimuli presented to the CI-simulation ear were processed according to the Ideal strategy. In the second session, participants completed the same set of monaural and binaural conditions but when stimuli in the CI-simulation ear were processed according to the Realistic strategy (main experiment) or the Shifted strategy (additional experiment). Monaural SRTs were measured at the start of each session and were used to determine the SNRs with which to construct the monaural and binaural fixed-SNR test conditions that followed. Monaural test conditions were included for two reasons: (1) to confirm that monaural performance was close to the level predetermined by the SRT, for example, stimuli presented to the NH ear at NH50 were expected to produce an accuracy of 50% correct on average; (2) to provide monaural comparators to the binaural test conditions, which were measured under the same experimental conditions. In the binaural test conditions, the SNR at the NH ear was fixed at NH50, whereas the SNR at the CI-simulation ear either supported superior monaural performance compared with the NH ear (CI71) or supported similar performance (CI50).

A total of 50 trials were presented in each monaural and binaural condition. Pilot testing suggested that presenting trials in blocks of 10 trials or fewer minimized differential learning effects across the conditions. Accordingly, the 50 trials in each condition were presented in 5 blocks of 10 trials. The order of blocks was randomized with the constraint that two blocks from the same condition could not be presented sequentially. Performance in each individual condition was measured as the percentage of trials on which all three key words were reported correctly.

Binaural integration advantages were calculated as the difference in performance between binaural conditions and those monaural conditions in which listeners only had access to the CI-simulation ear. When measured in this way, an improvement in performance under binaural conditions represented a benefit from the addition of the NH ear. Any such improvements were therefore attributed to integration rather than better-ear listening as the NH ear was constrained experimentally to provide levels of monaural performance that did not exceed the CI-simulation ear and provided a copy of the speech information at a less-favorable SNR. Thus, binaural integration advantages represented benefits that were not achievable simply by listening using the better-ear only, whether defined based on monaural performance or SNR.

### Training

Before estimating the SRT in the NH ear, participants completed a block of 15 trials at an SNR of 3 dB and a block of 15 trials at an SNR of −6 dB. Before estimating SRTs in the CI-simulation ear, three training blocks of 15 trials were completed in which the SNR was progressively made more adverse (speech-alone, 9-dB SNR, 0-dB SNR). Before completing the monaural and binaural conditions, participants completed a block of 15 trials in each binaural condition.

## RESULTS

### Speech-Reception Thresholds

**Fig. 2.**
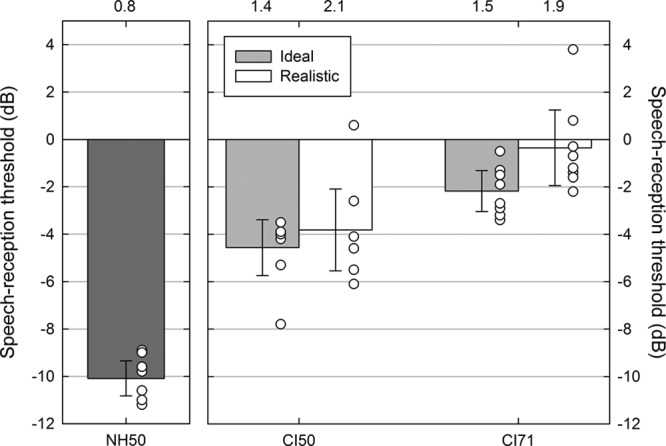
Mean (bars) and individual (symbols) speech-reception thresholds for the NH ear alone at 50% correct (NH50), the CI-simulation ear alone at 50% correct (CI50), and the CI-simulation ear alone at 71% correct (CI71) in the main experiment. Thresholds for the CI-simulation ear alone are shown for the Ideal (light gray bars) and Realistic (white bars) processing strategies. Error bars indicate 95% confidence intervals, and standard deviations are shown above the graph. CI indicates cochlear implant; NH, normal hearing.

Figure 2 shows the mean and individual SRTs measured in the NH ear and in the CI-simulation ear for the Ideal and Realistic processing strategies in the main experiment. With the NH ear alone, participants achieved an accuracy of 50% correct at an SNR of −10.1 dB (95% confidence interval, −10.8 to −9.3). The mean threshold for the NH ear alone was significantly lower (better) than the lowest CI-simulation ear SRT (CI50 Ideal, mean difference 5.5 dB, 95% confidence interval 4.6 to 6.5) [*t*(7) = 13.8, *p* < 0.001]. This disparity between the NH and the CI-simulation ears reflected the limitations of the CI simulations in conveying useful aspects of signals that aid the perception of speech in noise such as temporal fine structure (Moore 2008) and high-rate modulations in the temporal envelope (Stone et al. 2008).

With the CI-simulation ear alone, SRTs appeared to vary as a function of both difficulty (50% versus 71%) and processing strategy. The SNR required to achieve an accuracy of 50% correct was similar for the Ideal (mean −4.6 dB, 95% confidence interval −5.7 to −3.4) and Realistic (mean −3.8 dB, 95% confidence interval −5.5 to −2.1) processing strategies. The SNR required to reach 71% correct was numerically lower (better) for the Ideal strategy (mean −2.2 dB, 95% confidence interval −3.0 to −1.3) than for the Realistic strategy (mean −0.4 dB, 95% confidence interval −1.9 to 1.2).

**Fig. 3.**
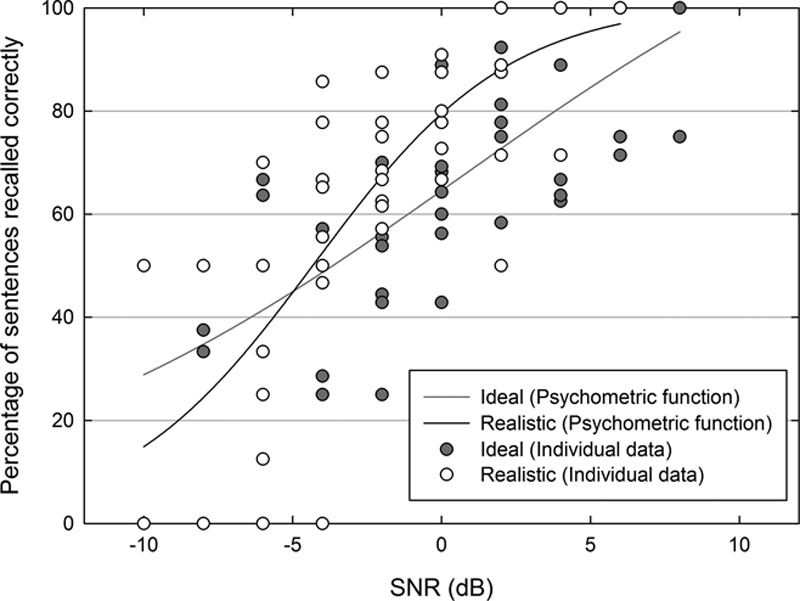
Psychometric functions showing the percentage of sentences for which all three key words were reported correctly as a function of SNR for the Ideal (solid gray line) and Realistic (solid black line) processing strategies. Data are extracted from the adaptive runs in the main experiment that estimated the Ideal (gray symbols) and Realistic (white symbols) CI71 thresholds. SNR indicates signal to noise ratio.

A repeated measures analysis of variance on the CI-simulation ear SRTs confirmed a significant effect of accuracy level (50% versus 71%) [*F*(1,7) = 164.1, *p* < 0.001] and a significant interaction between accuracy level and processing strategy (Ideal versus Realistic) [*F*(1,7) = 6.4, *p* < 0.05]. The main effect of processing strategy was not significant [*F*(1,7) = 4.5, *p* = 0.07]. Post hoc comparisons on the interaction confirmed that strategy affected CI71 SRTs [*t*(7) = 2.8, *p* < 0.05] but not CI50 SRTs [*t*(7) = 1.2, *p* > 0.05]. Participants therefore appeared to be less tolerant of noise when listening to the Realistic simulation compared with the Ideal simulation when also required to report what was said to a high degree of accuracy. This suggestion was supported by the presence of a steeper underlying psychometric function for the Realistic strategy (7.7% correct per dB SNR) compared with the Ideal strategy (4.1% correct per dB SNR) estimated by fitting a three-parameter sigmoidal function to the data extracted from the CI71 adaptive runs (Fig. 3).

The SRTs corresponding to 50% correct in the additional experiment were similar to those from the main experiment in both the NH ear (mean −9.5 dB, 95% confidence interval −10.6 to −8.4) and the CI-simulation ear (Ideal mean −3.9 dB,95% confidence interval −5.6 to −2.1; Shifted mean −4.2, 95% confidence interval −6.2 to −2.2). Unlike the main experiment, however, 71% SRTs were similar for both processing strategies (Ideal mean −1.1 dB, 95% confidence interval −2.8 to 0.7; Shifted mean −1.0, 95% confidence interval −2.7 to 0.8) and were not influenced by processing strategy [*t*(11) = −0.13, *p* > 0.05].

### Monaural Performance

Monaural performance was measured as the percentage of sentences on which all three key words were reported correctly and is listed in the left panel of Table 2. Performance levels with the NH ear at NH50 and with the CI-simulation ear at CI50 were numerically close to and not significantly different from an accuracy of 50% correct in both sessions and across both experiments. This finding also held for performance with the CI-simulation ear at CI71, which was numerically close to and not significantly different from the estimated level of 71%. As expected, performance levels were close to but not numerically identical to the levels estimated by the adaptive procedures but left room for improvement in the binaural conditions.

**TABLE 2.**
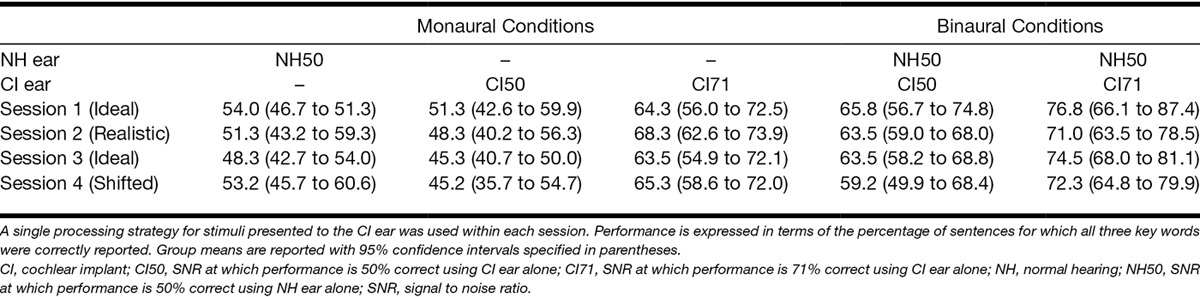
Summary of performance levels in the monaural and binaural listening conditions constructed using predetermined SNRs administered across the two sessions of the main experiment (sessions 1 and 2) and of the additional experiment (sessions 3 and 4)

### Binaural Performance

**Fig. 4.**
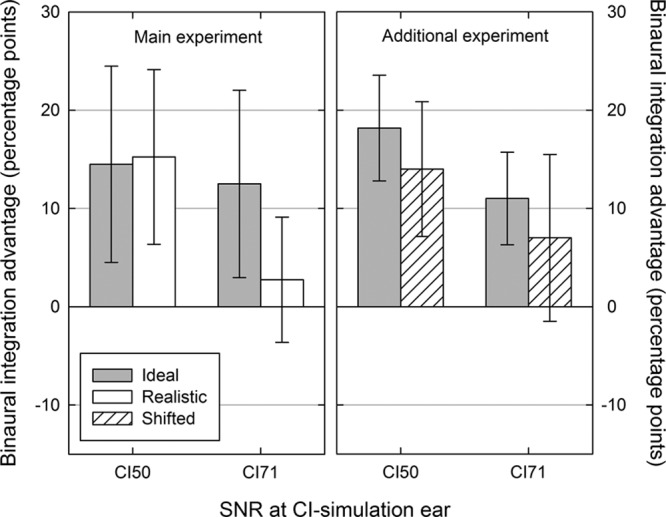
Mean binaural integration advantages for the Ideal (gray bars), Realistic (white bars), and Shifted (striped bars) processing strategies in the main experiment (left panel) and in the additional experiment (right panel). Binaural integration advantages were calculated as the change in the percentage of sentences recalled correctly when listening binaurally relative to listening monaurally using the CI-simulation ear alone (right panel). Error bars indicate 95% confidence intervals. CI indicates cochlear implant; SNR, signal to noise ratio.

Performance in the binaural conditions is listed in the right panel of Table 2. Binaural performance levels were always similar to or significantly better than the associated monaural conditions using either the NH or the CI-simulation ear. Binaural integration advantages are listed in Table 3 and shown in Figure 4 and were assessed relative to the CI-simulation ear alone in the CI50 and CI71 conditions. Advantages calculated in this way reflected the benefits arising from the additional use of the NH ear that always had a more adverse SNR and whose monaural performance was constrained not to exceed that of the CI-simulation ear. Evidence of a significant binaural integration advantage was found when the CI-simulation ear supported a similar level of performance (CI50) for both the Ideal strategy [*t*(7) = 3.4, *p* < 0.05] and the Realistic strategy [*t*(7) = 4.1, *p* < 0.01]. However, when the CI-simulation ear supported a superior level of performance (CI71), a binaural integration advantage was apparent only for the Ideal strategy [*t*(7) = 3.1, *p*< 0.05] and not for the Realistic strategy [*t*(7) = 1.0, *p* = 0.34].

**TABLE 3.**
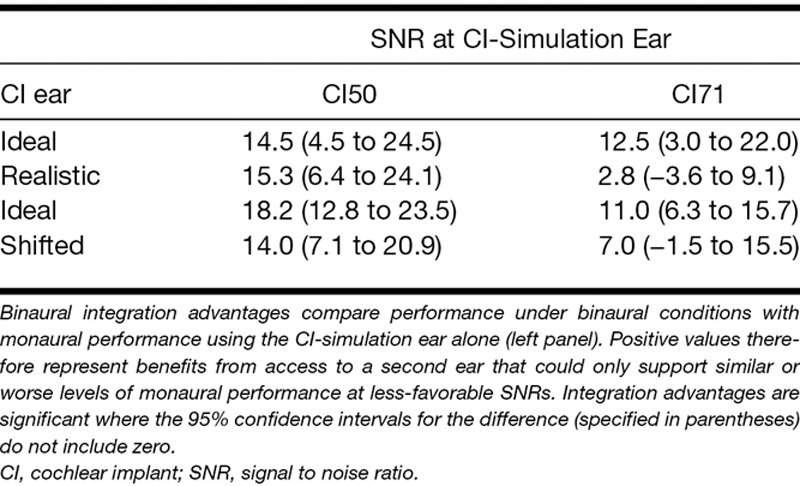
Summary of the binaural integration advantages observed across the different processing strategies in both the main experiment (top two rows) and the additional experiment (bottom two rows)

The additional experiment examined whether this difference between Realistic and Ideal strategies was a particular result of combining frequency shifting and compression rather than that of either effect alone by shifting the center frequency of each Ideal output filter basally by 3 mm (Shifted processing). The evidence for binaural integration advantages was similar to the main experiment (Table 3; Fig. 4). Significant binaural integration advantages were observed when the CI-simulation ear supported a similar level of monaural performance (CI50) both for the Ideal [*t*(11) = 7.4, *p* < 0.001] and Shifted [*t*(11) = 4.5, *p* < 0.001] processing strategies. When the CI-simulation ear supported a superior level of monaural performance (CI71), the pattern of results was similar to the main experiment, in that binaural integration was apparent when the delivery of spectral information was matched between the ears [Ideal strategy, *t*(11) = 5.1, *p* < 0.001] but not when a mismatch between the ears was introduced [Shifted strategy, *t*(11) = 1.8, *p* > 0.05].

**Fig. 5.**
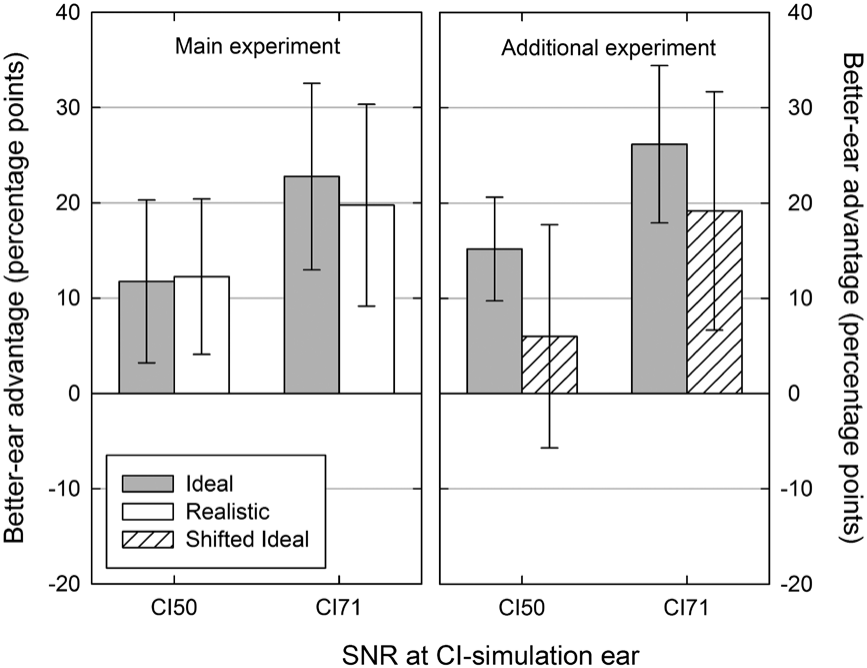
Mean better-ear advantages for the Ideal (gray bars), Realistic (white bars), and Shifted (striped bars) processing strategies in the main experiment (left panel) and additional experiment (right panel). Better-ear advantages were calculated as the change in the percentage of sentences recalled correctly when listening binaurally relative to listening monaurally using the normal-hearing ear alone. Error bars indicate 95% confidence intervals. CI indicates cochlear implant; SNR, signal to noise ratio.

To confirm that listeners could engage in better-ear listening and to assess whether better-ear benefits were also disrupted by a mismatch between the ears, binaural performance was also compared with monaural performance levels when using the NH ear alone. Measured in this way, any advantage derived from the additional use of the CI-simulation ear could be attributable to the fact that the second ear always provided a copy of the speech at a more favorable SNR and therefore were interpreted not as evidence for better-ear effects rather than integration. These “better-ear advantages” were found for both the Ideal and the Realistic strategies when the CI-simulation ear supported a similar level of monaural performance (CI50) and a superior level of monaural performance (CI71) compared with the NH ear (Table 4; Fig. 5).

**TABLE 4.**
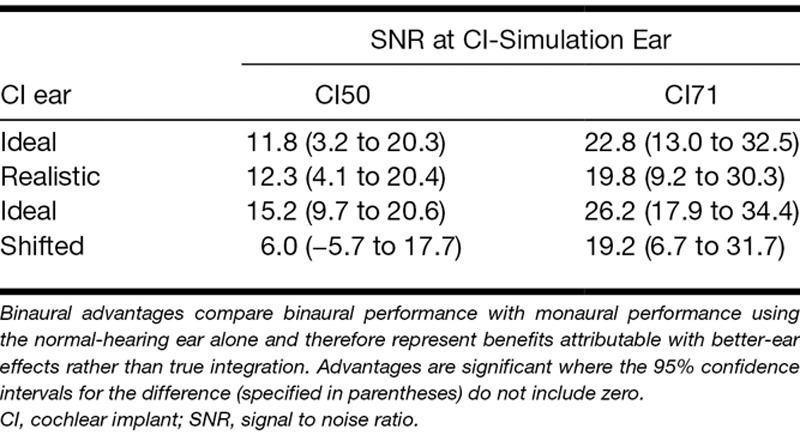
Summary of the better-ear advantages observed across the different processing strategies in both the main experiment (top two rows) and the additional experiment (bottom two rows)

A repeated measures analysis of variance on the better-ear advantages in the main experiment confirmed a main effect of CI-simulation ear SNR (CI50 versus CI71) [*F*(1,7) = 13.5, *p* < 0.01] but found no effect of strategy (Ideal versus Realistic) [*F*(1,7) = 0.08, *p* = 0.79] and no interaction [*F*(1,7) = 1.4, *p* = 0.23]. A similar result was found in the additional experiment with a significant main effect of CI-simulation ear SNR [*F*(1,11) = 17.6, *p* < 0.001] but not effect of strategy [*F*(1,11) = 2.9, *p* = 0.12] and no interaction [*F*(1,11) = 0.24, *p* = 0.64]. Thus, the additional use of the CI-simulation ear improved speech perception by providing access to a copy of the speech signal at a more favorable SNR than in the NH ear, and these better-ear effects did not appear to be disrupted by a mismatch in the delivery of spectral information between the two ears.

## DISCUSSION

This study measured the capacity of listeners to integrate information from an NH ear, with information from the contralateral ear that had been degraded spectrally and temporally to simulate a CI. The study also assessed whether this binaural integration may be disrupted by a mismatch in the delivery of spectral information between the ears arising from a misalignment in the mapping of frequency to place in the CI-simulation ear. The results suggested that in the absence of a mismatch, benefits to speech understanding in noise from binaural integration could be achieved both when two ears supported a similar level of monaural performance (NH50-CI50) and when the CI-simulation ear supported a superior level of monaural performance (NH50-CI71). A mismatch in the delivery of spectral information between the ears only appeared to disrupt binaural integration in the latter situation, that is, when the CI-simulation ear supported a superior level of performance on its own compared with the NH ear.

Performance across the binaural conditions was found to be either as accurate as or significantly more accurate than performance when using either the CI-simulation ear or the NH ear alone. This observation has also been made previously in evaluations of patients with a unilateral deafness after implantation. Aside from providing benefit by overcoming the head-shadow effect, Arndt et al. (2011) found that using the CI ear did not impair SRTs even when the SNR was less favorable at the implanted ear. Although the results of that study did not provide direct evidence for binaural integration, use of the CI did reduce self-reported listening difficulty in many everyday situations. Other studies have noted a numerical improvement (Jacob et al. 2011) or degradation (Vermeire & Van de Heyning 2009) in SRTs associated with CI use when the SNR is similar or worse than that at the NH ear, but none has reported a significant change in either direction under such listening conditions. The evidence from those early observational studies and from the present experiments therefore suggests that the provision of two-eared hearing in unilateral deafness can be beneficial to speech perception in noise and does not appear to interfere with speech perception even if signals from the two ears cannot be integrated.

Evidence of binaural integration was observed when the two ears supported a similar level of performance (NH50-CI50). Benefit from integration persisted under these conditions even when a mismatch was induced using either the Realistic or the Shifted processing strategies, unlike the integration benefit observed in the NH50-CI71 condition. The magnitude of the average binaural integration benefit appeared to be larger when the difference in monaural performance was smaller (compare CI50 and CI71 in Fig. 4), despite the absence of ceiling effects (Table 2). A relationship between binaural benefit and interaural functional asymmetry has been observed in CI users with limited residual hearing in whom greater benefit from listening binaurally was associated with a smaller difference between the monaural speech perception of their implanted and nonimplanted ears (Yoon et al. 2015). Although the size of the average binaural integration benefit in the present study was numerically larger in the NH50-CI50 condition compared with the NH50-CI71 condition, the difference was not statistically significant both in the main experiment [*F*(1,7) = 3.6, *p* > 0.05] and the additional experiment [*F*(1,11) = 4.1, *p* > 0.05]. A post hoc power calculation§ suggested that both experiments in the present study had sufficient power to detect effects of this size (main experiment: partial η^2^ = 0.34, achieved power 93%; additional experiment: partial η^2^ = 0.27, achieved power 97%). Therefore, if generalizable to unilaterally deaf CI users, the results of the present study would suggest that the size of the benefit from binaural integration does not depend on the degree of asymmetry in the monaural function of their two ears. However, the differential effects of introducing a mismatch in the NH50-CI50 and NH50-CI71 conditions suggest that integration may be more robust and less sensitive to a mismatch where the monaural performance of the two ears is similar.

One possible explanation for the lack of binaural integration in the NH50-CI71 condition when a mismatch between the ears was introduced is that integration was limited by ceiling effects. However, monaural performance in the CI-simulation ear at this SNR (CI71) was similar with and without a mismatch (Table 2, CI71), and binaural integration was observed when a mismatch was not present (Table 3, CI71 Ideal). Alternatively, it may be argued that binaural integration is not possible when information is spectrally misaligned between the ears. However, evidence for binaural integration was observed in the presence of a mismatch in the NH50-CI50 condition, despite the available information in the CI-simulation ear being more degraded (i.e., presented at a less-favorable SNR) compared with the NH50-CI71 condition.

Another possible explanation for the absence of evidence for integration in the NH50-CI71 condition when a mismatch was present is simply that there was an additional cost, perhaps in terms of processing load or perceived effort, in integrating spectrally mismatched information binaurally. Listeners may therefore have adopted a “better-ear” listening strategy in the NH50-CI71 condition as, unlike the NH50-CI50 condition, an improvement in performance over the NH ear alone could be achieved by simply attending to the CI-simulation ear, which supported more accurate performance when listening monaurally.

If the lack of binaural integration advantage in the mismatched NH50-CI71 condition reflected an inability to integrate, that effect could be attributed to one of two features of the Realistic processing strategy that gave rise to the mismatch, namely: (1) the delivery of spectral information to sites in the cochlea with a higher characteristic frequency resulting from the simulation of a plausible insertion depth (frequency shift); and (2) the delivery of a wide range of spectral information to a neural population with a smaller frequency range reflecting both the active length of contemporary CI electrode arrays and the wide input frequency range of speech processors applied by default (frequency compression). The additional experiment that induced a mismatch between the ears by misaligning the input and output filters in the CI-simulation ear while maintaining the simulated active length (Shifted processing; Table 1) produced a similar pattern of effects (Tables 2 and 3; Fig. 4) and confirmed that binaural integration can also be disrupted through a mismatch induced through frequency shifts in the absence of frequency compression. If the results of these simulations can be extrapolated to CI users, they would suggest that even if the input frequency range of a CI is adjusted to approximate the extent of characteristic frequencies within the nerve population being stimulated, difficulties with binaural integration may still persist unless each electrode delivers information at or close to the characteristic frequencies of the nerves it stimulates.

Although the present methodology controlled for monaural performance when assessing binaural benefit in different processing conditions, the SNR that was necessary to achieve the specified monaural performance level was free to vary with processing strategy. Listeners required a more favorable SNR to reach 71% correct using the CI-simulation ear alone with the Realistic strategy than with the Ideal strategy (right-hand side of Fig. 2). The selective disruption of binaural integration in the NH50-CI71 condition when a mismatch was introduced could therefore be attributed to a change in SNR in the CI-simulation ear rather than to an effect of processing strategy. However, the results of the additional experiment did not support this hypothesis. SRTs for the monaural CI71 condition were similar regardless of the processing strategy (Shifted mean −1.0 dB, 95% confidence interval −2.7 to 0.8; Ideal mean −1.1, 95% confidence interval −2.8 to 0.7), but binaural integration was still observed to be disrupted selectively by the presence of a mismatch in the NH50-CI71 condition (right-hand side of Fig. 4). Taken as a whole, the results suggest that the disruption of binaural integration in both experiments may have been driven by the introduction of a mismatch in the delivery of spectral information between the ears rather than from any changes in SNR.

A limitation of the present study is that it used vocoder processing to simulate the information conveyed through a CI. Simulations allow for characteristics such as the depth of insertion or frequency-to-place mapping to be manipulated experimentally in a controlled and consistent manner across participants. Vocoder simulations, such as those used here, typically use broad analysis and output filters to approximate the fact that many implant users have poor frequency resolution equivalent to about eight channels of spectral information (Niparko 2009). However, vocoder simulations are still presented to NH ears and therefore do not accurately simulate features of electrical stimulation such as a wide spread of excitation or the stimulation of cochlear sites located on the opposite side of the modiolus (“cross-turn” stimulation; Cohen et al. 2003).

A further limitation of using vocoder simulations is that, even after extensive training, NH listeners are unlikely to achieve the level of adaptation and learning exhibited by CI users after months and years of implant use. For example, unilaterally deaf CI users may be able to gradually adapt to timing differences between electric and acoustic information that can otherwise inhibit binaural fusion (Aronoff et al. 2015). Long-term follow-up of unilaterally deaf CI users have also demonstrated that the head-shadow effect and the binaural benefits of summation and squelch continue to increase in size 12 and 18 months after implantation (Gartrell et al. 2014). If the results of the present simulation study can be generalized to CI users, it is likely that they may therefore underestimate the capacity of unilaterally deaf CI users to integrate speech information binaurally.

It is also possible that the present results overstate the effects of a mismatch in the delivery of spectral information between the ears on binaural integration. Although studies have found that NH listeners do adapt to spectrally shifted speech after relatively short-term exposure (Rosen et al. 1999; Fu et al. 2005), studies using pitch-matching techniques with CI users suggest that adaptation to misalignments between frequency and cochlear place may take an extended period of time and reflect considerable plasticity in the cortical processing of electric information (Reiss et al. 2008). Studies of unilaterally deaf CI users also suggest that the nature and degree of the frequency-to-place misalignment that gives rise to the mismatch between the ears can be difficult to predict based on cochlear place alone, as assumed in the present study. Although some studies have observed pitch percepts that are compatible with cochlear place maps (Carlyon et al. 2010), others have observed pitches that were lower than predicted (Dorman et al. 2007). The degree of adaptation over time may also depend on the size of the misalignment. Vermeire et al. (2015) examined changes in the acoustically matched pitch of electrodes over time in five unilaterally deaf CI users. Numerical changes in the perceived pitch of electrodes were observed 12 months after implantation but were not statistically significant. The authors suggested that this apparent lack of adaptation may be attributable to the fact that misalignment was minimized initially due to the use of longer electrode arrays. The limited number of studies that have characterized the perceived pitch of electrodes in unilaterally deaf CI users means that it is difficult to make assumptions about the size and time-course of any changes in the perceived pitch of electrical stimulation, or what their effect may be on electroacoustic integration.

If a mismatch in the delivery of spectral information between the ears does disrupt binaural integration in these patients, it is unclear whether it would be feasible and practical to allocate frequencies in the CI to reduce mismatch and aid binaural integration. The depth to which electrode arrays are inserted varies considerably across patients (Finley et al. 2008) and has been found to vary across cohorts of patients recruited at different implant centers even when the same electrode array had been used (Landsberger et al. 2015). As a result, a frequency-to-place misalignment would be expected to occur in many patients if a nonindividualized frequency-to-electrode allocation is used. Those CI users with deeper insertions and for which there is likely to be a larger misalignment have been found to have poorer outcomes, particularly when measured as the ability to understand sentences in noise (Yukawa et al. 2004). The likelihood of creating a misalignment could be reduced, at least in part, from the preoperative selection of electrode array length based on cochlear imaging (Venail et al. 2015). Postoperatively, individualized frequency-to-electrode allocations could possibly be derived from computerized tomography imaging (Noble et al. 2014) and informed by pitch-matching tasks (Carlyon et al. 2010; Schatzer et al. 2014; Vermeire et al. 2015). However, it is as yet unclear whether these modifications to clinical practice would yield sufficient benefits to justify the additional time and resources required to implement them.

In summary, the present experiments with NH listeners suggest that unilaterally deaf individuals who use a CI may have the capacity to integrate information from their implanted and NH ears but that such binaural integration may be disrupted by a mismatch in the delivery of spectral information between the ears arising from a frequency-to-place misalignment in their implanted ear. The lack of integration benefits observed in previous clinical studies may therefore be explained, in part, by the fact that the process of mapping input frequencies to electrodes in those studies did not account for the position of the electrode array within the cochlea. Perhaps encouragingly, the present simulation experiments suggest that integration may not be disrupted by a mismatch in all circumstances. Integration was found to be resistant to disruption when the SNR at the two ears differed by approximately 5 to 6 dB (NH50-CI50 condition). An interaural difference of this magnitude can plausibly be created in everyday situations by the acoustic shadow cast by the head across a wide range of frequencies (Moore 2003).

Integration benefits in unilaterally deaf CI users can be difficult to measure using free-field presentation due to the large difference in the working SNR of their NH and implanted ears. The present experimental paradigm, which controls for individual differences in monaural speech understanding in each ear, could be a useful tool for assessing binaural integration in future studies that seeks to evaluate outcomes in unilaterally deaf patients after implantation.

## ACKNOWLEDGMENTS

N.M. and P.T.K. designed the experiments. N.M. collected the data for the main experiment, and S.M. collected the data for the additional experiment. N.M. and P.T.K. analyzed the data and drafted the manuscript. All authors contributed to and approved the final version. The authors thank Alan Palmer for helpful discussions and Hala Al Taher for assistance with piloting the sentence test.

## Footnotes

* The simulation replaces informative temporal fine structure (Moore 2008) with uninformative temporal fine structure while largely preserving the temporal envelope, i.e., the slow changes in a stimulus’ amplitude over time (Eaves et al. 2011). In addition, the simulation provides eight channels of spectral information, which represents the approximate number of functional channels provided by a cochlear implant (Niparko 2009).

† The “length” of the simulated array corresponded to the distance between the lower edge of the most apical filter and the upper edge of the most basal filter in millimeters on the basilar membrane according to Greenwood’s function (Greenwood 1990).

‡ Imposing a constant basal shift of 3 mm on all channels has the effect of presenting spectral information to sites in the cochlea with a higher corresponding characteristic frequency and broader auditory filter width while maintaining the channel separation. As a result, spectral information that may have previously fallen into separate auditory filters may now fall within a single auditory filter, effectively reducing spectral resolution.

§ The post hoc power calculations determined whether the two experiments had sufficient power to detect a difference in the size of the binaural integration benefit between the NH50-CI50 and the NH50-CI71 conditions. To determine the effect size, the binaural integration advantages (see Fig. 4) were subjected to an analysis of variance with within-subject factors of condition (NH50-CI50 versus NH50-CI71) and processing strategy (main experiment: Ideal versus Realistic; additional experiment: Ideal versus Shifted). The post hoc power calculation was based on the observed size of the main effect of condition and performed using the G*Power software (Faul et al. 2007).

## References

[R1] Arndt S. Aschendorff A. Laszig R. Comparison of pseudobinaural hearing to real binaural hearing rehabilitation after cochlear implantation in patients with unilateral deafness and tinnitus. Otol Neurotol(2011)32 39 47 21068690 10.1097/MAO.0b013e3181fcf271

[R2] Aronoff J. M. Shayman C. Prasad A. Unilateral spectral and temporal compression reduces binaural fusion for normal hearing listeners with cochlear implant simulations. Hear Res(2015)320 24 29 25549574 10.1016/j.heares.2014.12.005 PMC4440320

[R3] Bess F. H. Tharpe A. M. An introduction to unilateral sensorineural hearing loss in children. Ear Hear(1986)7 3 13 3512353 10.1097/00003446-198602000-00003

[R4] Bishop C. E. Eby T. L. The current status of audiologic rehabilitation for profound unilateral sensorineural hearing loss. Laryngoscope(2010)120 552 556 20014322 10.1002/lary.20735

[R5] Carlyon R. P. Macherey O. Frijns J. H. Pitch comparisons between electrical stimulation of a cochlear implant and acoustic stimuli presented to a normal-hearing contralateral ear. J Assoc Res Otolaryngol(2010)11 625 640 20526727 10.1007/s10162-010-0222-7 PMC2975889

[R6] Christensen L. Richter G. T. Dornhoffer J. L. Update on bone-anchored hearing aids in pediatric patients with profound unilateral sensorineural hearing loss. Arch Otolaryngol Head Neck Surg(2010)136 175 177 20157065 10.1001/archoto.2009.203

[R7] Cohen L. T. Richardson L. M. Saunders E. Spatial spread of neural excitation in cochlear implant recipients: Comparison of improved ECAP method and psychophysical forward masking. Hear Res(2003)179 72 87 12742240 10.1016/s0378-5955(03)00096-0

[R8] Donaldson G. S. Chisolm T. H. Blasco G. P. BKB-SIN and ANL predict perceived communication ability in cochlear implant users. Ear Hear(2009)30 401 410 19390441 10.1097/AUD.0b013e3181a16379

[R9] Dorman M. F. Spahr T. Gifford R. An electric frequency-to-place map for a cochlear implant patient with hearing in the nonimplanted ear. J Assoc Res Otolaryngol(2007)8 234 240 17351713 10.1007/s10162-007-0071-1 PMC2441831

[R10] Eaves J. M. Summerfield A. Q. Kitterick P. T. Benefit of temporal fine structure to speech perception in noise measured with controlled temporal envelopes. J Acoust Soc Am(2011)130 501 507 21786915 10.1121/1.3592237

[R11] Faul F. Erdfelder E. Lang A. G. G*Power 3: A flexible statistical power analysis program for the social, behavioral, and biomedical sciences. Behav Res Methods(2007)39 175 191 17695343 10.3758/bf03193146

[R12] Finley C. C. Holden T. A. Holden L. K. Role of electrode placement as a contributor to variability in cochlear implant outcomes. Otol Neurotol(2008)29 920 928 18667935 10.1097/MAO.0b013e318184f492 PMC2663852

[R13] Firszt J. B., Holden L. K., Reeder R. M. (2012a). Cochlear implantation in adults with asymmetric hearing loss.. Ear Hear.

[R14] Firszt J. B., Holden L. K., Reeder R. M. (2012b). Auditory abilities after cochlear implantation in adults with unilateral deafness: A pilot study.. Otol Neurotol.

[R15] Fu Q. J. Nogaki G. Galvin J. J. III Auditory training with spectrally shifted speech: Implications for cochlear implant patient auditory rehabilitation. J Assoc Res Otolaryngol(2005)6 180 189 15952053 10.1007/s10162-005-5061-6 PMC2538336

[R16] Gartrell B. C. Jones H. G. Kan A. Investigating long-term effects of cochlear implantation in single-sided deafness: A best practice model for longitudinal assessment of spatial hearing abilities and tinnitus handicap. Otol Neurotol(2014)35 1525 1532 25158615 10.1097/MAO.0000000000000437 PMC4334463

[R17] Greenwood D. D. A cochlear frequency-position function for several species—29 years later. J Acoust Soc Am(1990)87 2592 2605 2373794 10.1121/1.399052

[R18] Hassepass F. Aschendorff A. Wesarg T. Unilateral deafness in children: Audiologic and subjective assessment of hearing ability after cochlear implantation. Otol Neurotol(2013)34 53 60 23202150 10.1097/MAO.0b013e31827850f0

[R19] Jacob R. Stelzig Y. Nopp P. Audiological results with cochlear implants for single-sided deafness. HNO(2011)59 453 460 21533601 10.1007/s00106-011-2321-0

[R20] Kitterick P. T. Bailey P. J. Summerfield A. Q. Benefits of knowing who, where, and when in multi-talker listening. J Acoust Soc Am(2010)127 2498 2508 20370032 10.1121/1.3327507

[R21] Köbler S. Rosenhall U. Horizontal localization and speech intelligibility with bilateral and unilateral hearing aid amplification. Int J Audiol(2002)41 395 400 12403607 10.3109/14992020209090416

[R22] Landsberger D. M. Svrakic M. Roland J. T. Jr. The relationship between insertion angles, default frequency allocations, and spiral ganglion place pitch in cochlear implants. Ear Hear(2015)36 e207 e213 25860624 10.1097/AUD.0000000000000163 PMC4549170

[R23] Levitt H. Transformed up-down methods in psychoacoustics. J Acoust Soc Am(1971)49 Suppl 2 467 5541744

[R24] Litovsky R. Y. Parkinson A. Arcaroli J. Spatial hearing and speech intelligibility in bilateral cochlear implant users. Ear Hear(2009)30 419 431 19455039 10.1097/AUD.0b013e3181a165be PMC2873678

[R25] McLeod B. Upfold L. Taylor A. Self reported hearing difficulties following excision of vestibular schwannoma. Int J Audiol(2008)47 420 430 18574780 10.1080/14992020802033083

[R26] Moore B. C. J. An Introduction to the Psychology of Hearing(2003)5th ed. Academic Press

[R27] Moore B. C. The role of temporal fine structure processing in pitch perception, masking, and speech perception for normal-hearing and hearing-impaired people. J Assoc Res Otolaryngol(2008)9 399 406 18855069 10.1007/s10162-008-0143-x PMC2580810

[R28] Moore T. J. Voice communication jamming research. In K. E. Money (Ed.), AGARD Conference Proceedings 311: Aural Communication in Aviation (AGARD)(1981)Neuilly-Sur-Seine, France: North Atlantic Treaty Organization, Advisory Group for Aerospace Research & Development pp. 2:1 2:6

[R29] Niparko J. K. Cochlear Implants: Principles & Practices(2009)Philadelphia, PA Lippincott Williams & Wilkins

[R30] Noble W. Gatehouse S. Interaural asymmetry of hearing loss, Speech, Spatial and Qualities of Hearing Scale (SSQ) disabilities, and handicap. Int J Audiol(2004)43 100 114 15035562 10.1080/14992020400050015

[R31] Noble J. H. Gifford R. H. Hedley-Williams A. J. Clinical evaluation of an image-guided cochlear implant programming strategy. Audiol Neurootol(2014)19 400 411 25402603 10.1159/000365273 PMC4305276

[R32] Pumford J. Benefits of probe-mic measures with CROS/BiCROS fittings. Hear J(2005)50 34 40

[R33] Reiss L. A. Gantz B. J. Turner C. W. Cochlear implant speech processor frequency allocations may influence pitch perception. Otol Neurotol(2008)29 160 167 18025998 10.1097/mao.0b013e31815aedf4 PMC4243703

[R34] Rosen S. Faulkner A. Wilkinson L. Adaptation by normal listeners to upward spectral shifts of speech: Implications for cochlear implants. J Acoust Soc Am(1999)106 3629 3636 10615701 10.1121/1.428215

[R35] Schatzer R. Vermeire K. Visser D. Electric-acoustic pitch comparisons in single-sided-deaf cochlear implant users: Frequency-place functions and rate pitch. Hear Res(2014)309 26 35 24252455 10.1016/j.heares.2013.11.003

[R36] Schleich P. Nopp P. D’Haese P. Head shadow, squelch, and summation effects in bilateral users of the MED-EL COMBI 40/40+ cochlear implant. Ear Hear(2004)25 197 204 15179111 10.1097/01.aud.0000130792.43315.97

[R37] Stone M. A. Füllgrabe C. Moore B. C. Benefit of high-rate envelope cues in vocoder processing: Effect of number of channels and spectral region. J Acoust Soc Am(2008)124 2272 2282 19062865 10.1121/1.2968678

[R38] Tharpe A. M. Sladen D. P. Causation of permanent unilateral and mild bilateral hearing loss in children. Trends Amplif(2008)12 17 25 18270175 10.1177/1084713807313085 PMC4111449

[R39] Vaerenberg B. Smits C. De Ceulaer G. Cochlear implant programming: A global survey on the state of the art. ScientificWorldJournal(2014)2014 501738 24688394 10.1155/2014/501738 PMC3932199

[R40] Venail F. Mathiolon C. Menjot de Champfleur S. Effects of electrode array length on frequency-place mismatch and speech perception with cochlear implants. Audiol Neurootol(2015)20 102 111 25678235 10.1159/000369333

[R41] Vermeire K. Van de Heyning P. Binaural hearing after cochlear implantation in subjects with unilateral sensorineural deafness and tinnitus. Audiol Neurootol(2009)14 163 171 19005250 10.1159/000171478

[R42] Vermeire K. Landsberger D. M. Van de Heyning P. H. Frequency-place map for electrical stimulation in cochlear implants: Change over time. Hear Res(2015)326 8 14 25840373 10.1016/j.heares.2015.03.011 PMC4524783

[R43] Wie O. B. Pripp A. H. Tvete O. Unilateral deafness in adults: Effects on communication and social interaction. Ann Otol Rhinol Laryngol(2010)119 772 781 21140638

[R44] Yoon Y. S. Shin Y. R. Fu Q. J. Binaural benefit with and without a bilateral spectral mismatch in acoustic simulations of cochlear implant processing. Ear Hear(2013)34 273 279 22968427 10.1097/AUD.0b013e31826709e8 PMC3525748

[R45] Yoon Y. S. Shin Y. R. Gho J. S. Bimodal benefit depends on the performance difference between a cochlear implant and a hearing aid. Cochlear Implants Int(2015)16 159 167 25329752 10.1179/1754762814Y.0000000101 PMC5847325

[R46] Yukawa K. Cohen L. Blamey P. Effects of insertion depth of cochlear implant electrodes upon speech perception. Audiol Neurootol(2004)9 163 172 15084821 10.1159/000077267

